# From neglected to notoriety: a review of Mpox clinical features, virology, epidemiology, treatment and prevention strategies

**DOI:** 10.1007/s10096-025-05242-1

**Published:** 2025-09-10

**Authors:** Clément Viguier, Pierre Delobel, François-Xavier Lescure, Simon Bessis, Jean-Michel Mansuy, Guillaume Martin-Blondel

**Affiliations:** 1 Department of Infectious and Tropical Diseases, Toulouse University Hospital, Toulouse, 31059 Cedex 9 France; 2 NEO-I3D Research Group, Toulouse University Hospital, Toulouse, France; 3 Toulouse Institute for Infectious and Inflammatory Diseases, INSERM UMR1291 - CNRS UMR5051 - Toulouse III University, Toulouse, France; 4 Department of Infectious and Tropical Diseases, APHP, Bichat – Claude Bernard Hospital, Paris, France; 5 IAME, INSERM UMR 1137, Université Paris Cite, Paris, France; 6 Biology of Viral Emerging Infections, INSERM UMR5308, Institut Pasteur, Lyon, France; 7 International Center for Infectiology Research (CIRI), Claude Bernard Lyon 1 University, Lyon, France; 8 Department of Virology, Toulouse University Hospital, Toulouse, France

**Keywords:** Mpox, Monkeypox virus, Epidemiology, Anti-viral treatment, Vaccination

## Abstract

**Purpose:**

This narrative review aims to provide an overview of current knowledge on mpox, emphasizing updated epidemiology and recent advances in treatment and prevention strategies, in light of the latest outbreaks.

**Methods:**

We searched PubMed and Google Scholar for publications on ‘Mpox’ and ‘Monkeypox’ up to June 5, 2025. Grey literature from governmental and health agencies was also accessed for outbreak reports and guidelines where published evidence was unavailable.

**Results:**

Recent outbreaks have redefined mpox epidemiology. Whereas previous regional outbreaks were mainly driven by zoonotic spillover with limited household transmission and often affecting children, more recent outbreaks have involved sustained human-to-human transmission. Such transmission has occurred among men who have sex with men for clade IIb and within heterosexual networks for clade Ib outbreaks, and more recently clade Ia outbreaks, primarily through sexual contact. Clinical features have also shifted toward more localized lesions, prominently in the anogenital area. While mpox is usually self-limited, severe cases may occur in pregnant women, young children, and immunocompromised individuals. Mpox management primarily relies on supportive care. In patients with severe mpox, or at risk of, tecovirimat was widely recognized as the first-line therapy, although it has failed to demonstrate its effectiveness in recent randomized controlled trials. The Modified Vaccinia Ankara vaccine (two-dose regimen) has shown a favorable safety profile and promising efficacy data in preventing clade IIb mpox, including immunocompromised individuals.

**Conclusion:**

Mpox has transitioned from a neglected zoonosis to a re-emerging global health threat. Sustained surveillance, robust and targeted public health interventions, and equitable access to diagnostics, vaccines, and antiviral treatments are critical to managing potential future mpox outbreaks.

## Introduction

Mpox (formerly known as monkeypox) is an emerging zoonosis caused by the monkeypox virus (MPXV), an orthopoxvirus closely related to the vaccinia and variola viruses, which affects both humans and a wide range of animals [1]. MPXVs are classified into two main clades evolving from a common ancestor: clade I (Central African clade), historically associated with more severe disease, and clade II (West African clade), historically associated with milder disease [2]. After its first isolation in 1958 in cynomolgus macaques shipped from Singapore to Copenhagen [3], followed by several outbreaks in captive monkeys in Western countries over the next decade, the first human case of MPXV infection was reported in 1970 in the Democratic Republic of the Congo (DRC) in a 9-month-old boy, the only member of his family without smallpox vaccination, presenting with a febrile, diffuse skin rash associated with painful cervical lymphadenopathy [4]. Subsequently, MPXV continued to circulate mainly in Central and West African countries through sporadic outbreaks, primarily related to animal-to-human transmission (primates, ruminants, rodents), but also limited human-to-human transmission [5]. Over the past decades, in the context of the gradual decline of herd immunity after the discontinuation of smallpox vaccination in 1980, the incidence of mpox and its geographic distribution have further increased due to various factors such as population movements and displacements, urbanization in rainforest areas, and the expansion of international trade, with the vast majority of cases related to clade I in Central Africa, mainly in the DRC, but also a major clade II outbreak in Nigeria in 2017, while West African countries previously only experienced sporadic mpox cases [6–8]. Mpox was first reported outside the African continent in 2003, with 71 human cases in the United States (US) linked to the importation of Gambian pouched rats from Ghana, followed by a few sporadic cases reported in other non-endemic countries, mainly related to travel or contact with animals from endemic areas [9, 10]. Despite this, mpox remained a neglected disease with limited related research.

Mpox finally generated a major international attention in 2022 after the rapid and massive community spread of a new virus belonging to the clade IIb lineage B.1 in non-endemic countries worldwide [11–13]. This global outbreak, declared by the World Health Organization (WHO) a Public Health Emergency of International Concern (PHEIC) in July 2022, had affected 122 countries by June 2023 (115 of which had never reported cases of mpox before) with a total of 87,972 cases diagnosed [14, 15]. It significantly differed from previous reported outbreaks, with a large majority of cases among men who have sex with men (MSM) and with sexual intercourse being the main route of transmission [11]. Currently, mpox remains a significant public health concern, primarily affecting African countries with the emergence of MPXV clade Ib from September 2023 in the DRC, subsequently spreading to other bordering African countries and then sporadically outside Africa from August 2024. This resurgence prompted a second declaration of PHEIC in August 2024 [16]. Meanwhile, MPXV clade IIb lineage B.1 continues to circulate at a low level worldwide, along with the rise of MPXV clade Ia in Central African countries [17, 18].

This review presents an overview of mpox, including its epidemiology, modes of transmission, clinical presentation, diagnostic strategy, prognosis, and an updated examination of available treatments and vaccines.

## Virology

MPXV is one of 14 DNA viruses belonging to the Orthopoxvirus genus, along with the vaccinia virus (used in the smallpox vaccine), the variola virus (agent of smallpox), the cowpox virus, and others including recently described viruses such as Borealpox virus. Orthopoxviruses are closely related genetically and antigenically [1]. As a result, there is cross-immunity between these viruses, which explains the relative protection conferred by smallpox vaccination against Mpox [19].

MPXV virions range from 200 to 250 nm with a 194–199 kb double-stranded genome encoding around 200 viral proteins [20].

Based on the sequence identity of strains historically isolated from various African countries, MPXVs were divided into two genetic clades, namely clade I and clade II, respectively isolated from Central (Congo basin) and West African regions, with approximately 0.5% inter-clade genomic sequence difference [2, 21]. Beyond geographic distribution, these two clades vary in severity, transmissibility, and clinical presentation. Clade I viruses have historically been associated with higher case-fatality rates (1–12%) than clade II (with case-fatality rates < 0.1-1%) [2, 8, 11, 22–24]. Genomic differences between these two clades primarily involve regions encoding key virulence genes, which may at least partially explain the difference in clinical severity [25]. For instance, a gene encoding a complement control protein found in MPXV clade Ia is absent in clade Ib and II viruses [26]. This protein may play a role in the immune evasion of MPXV by preventing the initiation of the complement pathway. An animal model study demonstrated reduced mortality and severity with clade Ia MPXV after deletion of this complement control protein [27].

With the emergence of MPXV in non-endemic regions during the 2022 outbreak, the WHO renamed the disease mpox and revised the clade nomenclature to reduce stigma and avoid any negative impacts on communities, trade, and animal welfare. The former Congo basin clade is now referred as clade I, and the West African clade as clade II, which includes two subclades—IIa and IIb—the latter comprising lineage B.1, responsible for the 2022 global outbreak [28].

## Transmission

Unlike smallpox, a strictly human pathogen, MPXV has a broad host range, including both animals and humans. Despite its name, monkeys are not the reservoir of MPXV; monkeys and humans are indeed considered incidental hosts [29]. Although the exact animal reservoir of MPXV remains unknown, it has been isolated from a wide range of rodents and non-primate animals in Central and West African rainforests (e.g., Gambian pouched rats, tree squirrels, or rope squirrels) [29–31].

Animal-to-human transmission occurs through contact with lesions or body fluids of infected animals, bites or scratches, or non-invasive contact with contaminated fomites, aerosols, or during wild meat handling [32–34]. Conversely, human-to-animal transmission has been suggested in only one case involving a domestic dog that may have been infected following contact with its infected owners in France [35]. The overall risk of reverse zoonosis is currently considered low, based on a United Kingdom (UK) surveillance study of 154 pets in households with confirmed mpox cases, none of which developed signs of infection [36].

Human-to-human transmission of MPXV primarily occurs via direct skin and mucosal contact, but can also occur through exposure to respiratory secretions, maternal-foetal transmission, percutaneous inoculation or indirect contact via contaminated fomites [37].

Direct and prolonged skin and mucosal contact played a pivotal role in MPXV transmission during the 2022 global outbreak. MPXV DNA was detected in skin lesion samples from nearly all infected individuals (over 90% during the first two weeks of illness), with this sample site presenting the highest viral loads, approximately two orders of magnitude higher than others (viral load 10^6^ vs. 10^4^ copies/ml) [38, 39]. In contrast, viral DNA was less frequently detected and at lower levels at other sample sites during the first week of symptoms, with DNA detected in 88% of saliva samples, 44–71% of rectal swabs, 37–77% of throat swabs, 22–27% of urine samples, 26% of nasopharyngeal swabs, and 8%−67% of semen samples [38, 40–42].

Replication-competent virus was successfully isolated from all these specimens, albeit more consistently from skin lesions. Regarding viral clearance, data retrieved from patients during the 2022 global outbreak show that MPXV DNA persists longest in skin lesions, with a median time from symptom onset of 23–25 days, compared to 11–16 days in oropharyngeal samples, and 15–16 days in rectal swabs [39, 43]. Importantly, replication-competent viruses were retrieved from receptive mucosal sites such as rectal and oropharyngeal samples, and could therefore be a source of infection, although at a lower frequency and with lower viral load than in skin lesions [38, 39]. Although semen has attracted attention as a potential vector for sexual transmission, viral loads were low in most studies, with only 1% of semen samples having a viral load above the limit of successful viral culture (i.e., 6.5 log^10^ copies per ml) in a Spanish cohort of 77 mpox clade IIb patients [39]. Moreover, viral clearance in semen occurred relatively quickly (median time 10.5–14 days) [39, 41, 42]. Combined with the fact that most cases during the 2022 outbreak involved lesions located in the anogenital and oropharyngeal regions, these data support the hypothesis that intimate skin contact—primarily through sexual contact—was a major driver of the 2022 mpox outbreak. Accordingly, mpox has increasingly been recognized as a sexually transmitted infection (STI) [44]. However, the designation of mpox as an STI should not overshadow the possibility of non-sexual transmission in other close-contact settings, including caregiving and shared household environments [37].

Percutaneous transmission of MPXV has mainly been described after needle-stick injuries with material used to collect skin lesions. This mode of transmission has mainly been described among healthcare workers, including laboratory technicians, but also outside healthcare settings such as tattoo and piercing salons [45, 46].

Respiratory transmission has not been considered a major contributor in previous mpox outbreaks. It could occur when large shedding of viral particles from oral and pharyngeal mucosal lesions are disseminated through respiratory droplets to the nasal or oral mucosa of healthy individuals. Although this mode of transmission has been demonstrated in non-human primate and prairie dog models, at least for clade I MPXV, there is currently no known mpox outbreak in people with shared space without evidence of other direct contact, particularly skin contact [47–49]. For instance, a survey of 113 cases of MPXV infection on 221 commercial flights failed to identify any secondary cases [50]. Similarly, no secondary cases have been reported in contact-tracing studies conducted in collective or residential settings such as schools or penitentiary institutions [51, 52].

Although rarely documented, vertical transmission of MPXV to the foetus can occur, as demonstrated by several cases in the DRC between 2007 and 2011 [53]. No maternal-foetal transmission has been reported during the 2022 global outbreak [54].

Notably, growing evidence suggests that asymptomatic individuals may contribute to mpox spread. In a study conducted in 2022 at a French sexual health clinic, 13 out of 200 asymptomatic MSM (6.5%) tested positive for MPXV DNA in rectal swabs [55]. Similar findings were reported in a 2022 study conducted at a Belgian sexual health clinic, involving 224 men, with MPXV DNA detected in rectal and oral swabs from three individuals who remained asymptomatic over a 21–37-day period. Replication competent virus was isolated from two of these three cases, further supporting the potential for asymptomatic transmission [56]. Furthermore, presymptomatic individuals may contribute to a greater extent to MPXV spread, as demonstrated by a UK modelling study using contact tracing data and symptom onset timelines, estimating that up to 53% of MPXV transmissions may have occurred between 1 and 4 days before the onset of the first lesions [57].

## Epidemiology

The first human mpox case was reported in 1970 in the DRC [4]. Between 1970 and 1980, several sporadic outbreaks subsequently emerged in West and Central Africa, with an estimated 60 suspected cases, primarily in children, mainly in the rainforest regions of the DRC [58]. Until 2017, most mpox outbreaks occurred in Central Africa and were associated with clade I. To date, the DRC remains the most affected country globally, being the only one to have reported cases without interruption for 50 years. Data from the DRC national surveillance system over the past decades reveal a progressive increase in incidence of the disease, from 2.97 per 100,000 people in 2010 to 11.46 per 100,000 people in 2023, along with its progressive geographic expansion into previously unaffected regions [59].

In 2017, an outbreak linked to MPXV clade II lineage A.1 emerged in Nigeria, that had been free of reported mpox cases for almost 40 years. A total of 183 suspected or confirmed cases were reported, making it one of the largest outbreaks in Africa at the time [5, 60]. Until then, most cases in African countries were believed to result from multiple independent zoonotic spillovers, followed by short human-to-human transmission chains. The rising incidence in endemic areas and mpox’s re-emergence in Nigeria were attributed to the cessation of smallpox vaccination in 1980, bushmeat consumption, and urban expansion into rainforest areas [8, 61, 62]. However, unlike clade Ia viruses, clade IIb lineage A.1 viruses involved in the 2017 Nigerian epidemic displayed prolonged human-to-human transmission genetic signatures (APOBEC3), suggesting different transmission networks from previous epidemics. Similar genetic signatures were subsequently found in clade Ib and IIb lineage B.1 viruses [63, 64].

Since 2003, sporadic cases and minor outbreaks have been reported in non-endemic countries (including the US, Singapore, Israel and the UK), mainly in individuals returning from or exposed to infected animals exported from endemic areas [9, 10, 65–68]. Before 2022, the largest outbreak outside endemic areas occurred in the USA in 2003, involving 71 individuals, none of whom died. Investigations revealed that these cases were related to contact with prairie dogs infected through exposure to Gambian giant rats imported from Ghana [9, 10].

**Fig. 1:**
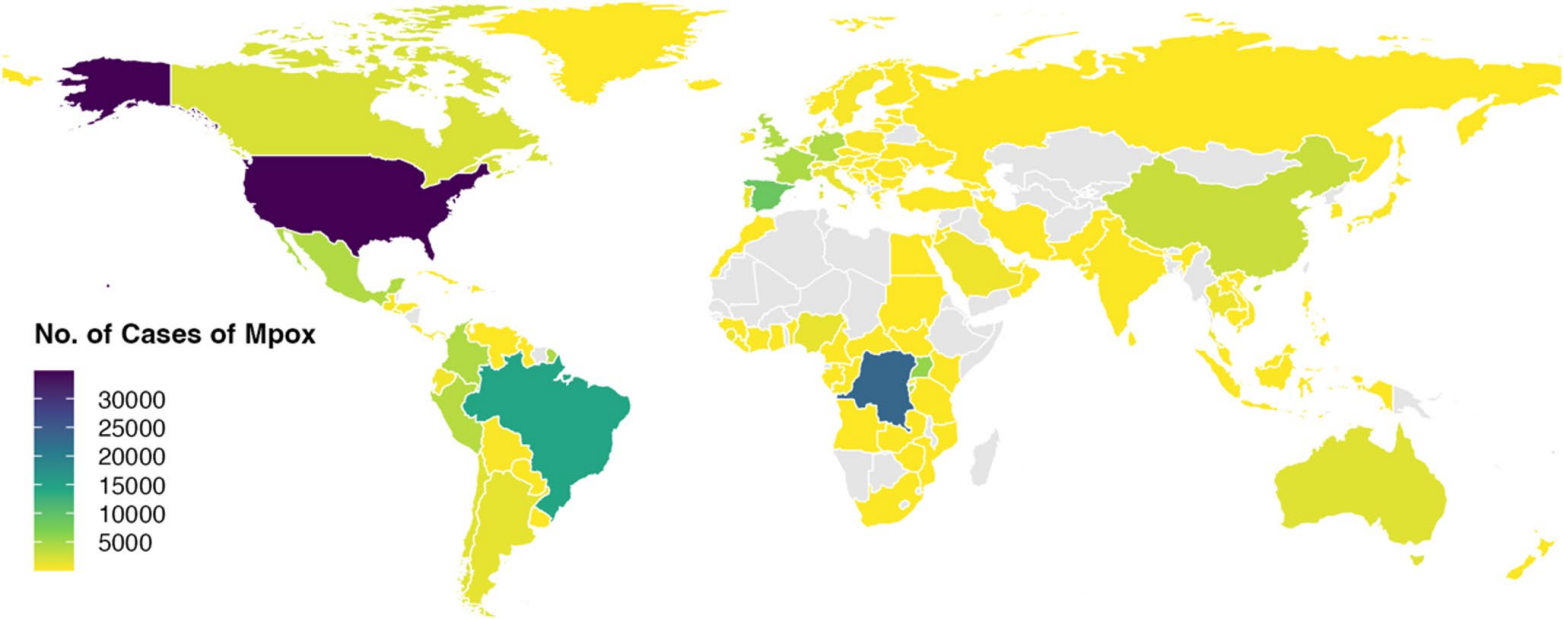
Geographic distribution of confirmed Mpox cases (all clades considered) from 01 January 2022 to 01 June 2025. A total of 142,212 confirmed Mpox cases (all clades considered) were reported globally from January 1, 2022, to June 1, 2025. The vast majority of cases were attributable to clade IIb, lineage B.1. The top three countries with the highest number of confirmed cases were the United States (*n* = 34,859), the Democratic Republic of the Congo (*n* = 23,280), and Brazil (*n* = 14,306). Data source: World Health Organization (https://worldhealthorg.shinyapps.io/mpx_global/#sec-down). Map generated with RStudio software (v2024.4.2.764). Grey areas indicate countries with no cases reported to the WHO

In early May 2022, the first mpox cases were reported to the WHO by the UK Health Security Agency, quickly followed by Portugal and Spain [69–71]. The number of cases rapidly increased worldwide, prompting the WHO to declare the mpox outbreak a PHEIC from July 2022 to May 2023 [14]. Investigations revealed that most of the early reported cases of mpox had not returned from endemic areas, but were linked to a LGBT + pride event held on Gran Canaria, Spain [71–73]. By mid-May, new mpox cases in all affected countries were primarily acquired locally, marking a shift from travel-associated cases to community transmission [73]. By June 2023, 122 countries worldwide, of which 115 had never declared mpox cases before, had reported 87,972 cases with 147 deaths [74]. This outbreak was caused by the new variant clade IIb lineage B.1, descending from lineage A.1 involved in the 2017–2019 Nigerian outbreak [75]. The 2022 outbreak is the largest mpox outbreak ever documented, both in terms of magnitude and global dissemination, and it significantly contrasts with previously reported outbreak. An analysis of 82,807 confirmed cases of mpox reported by WHO Member States through the global surveillance system up to 29 January 2023 showed an unusually high proportion of males (96.4%) with a median age of 34 years (IQR 29–41) [76]. Among 29,854 men with known sexual orientation, 86.9% self-identified as MSM. Additionally, almost half of the cases with known HIV status were HIV-positive (48.0%, 16,961/35,329) according to global surveillance data, and 74.2% of HIV-negative individuals were using pre-exposure prophylaxis (PrEP) for HIV in a UK cohort of 842 mpox patients [77]. Cohort data published to date indicate high-risk sexual behavior in a large proportion of patients: 31.8% of UK patients reported 10 or more sexual partners in the past 3 months, 16–29% were diagnosed with a concurrent STI, and 54% had a history of STI in the past 12 months [11, 77–79]. Combined with the non-usual lesion distribution, frequently in the genital, anal and perianal regions, and the fact that MPXV DNA was found in the seminal fluid of infected individuals, these findings suggest that the 2022 global outbreak was primarily driven by sexual transmission within MSM networks engaging in high-risk sexual behaviors [11, 80]. Within a few months, weekly cases number declined sharply worldwide, probably as a result of public health measures — including prompt case identification and isolation, vaccination of at-risk individuals, as well as behavioral changes with a reduction in high-risk sexual behaviors — and the immunity acquired in previously infected patients [18, 81, 82]. However, there is still a low-level circulation of mpox clade IIb lineage B.1 worldwide, with to date over 100,000 reported cases and 220 deaths [18] (Fig. 1).

In September 2023, clade Ib MPXV emerged in South Kivu, a region of eastern DRC previously unaffected by mpox. This new lineage is distinct from prior clade I MPXV strains identified in the DRC, now referred to as clade Ia [63]. Initial reports from the DRC indicated that, unlike previous clade Ia outbreaks, most cases involved adults presenting with moderate symptoms, predominantly genital lesions, and apparent human-to-human transmission via sexual contact [83]. These findings were later confirmed in a large prospective cohort of 510 clade Ib-infected patients, all residing in South Kivu [84]. Mpox predominantly affected adolescents and young adults, 48% of whom were female. The median age was 22 years (IQR 18–29), with children under 15 years accounting only for 21% of cases and those under 5 years for 3%. Patients typically presented with moderate symptoms. In adults, lesions were primarily localized to the genital area, whereas children had more extra-genital involvement. In-hospital mortality was low (0.5%), with two deaths reported among children under 5 years of age. Exposure data indicate that transmission was essentially mediated by sexual or intimate contact between adults, then spreads to households, particularly among children.

**Fig. 2:**
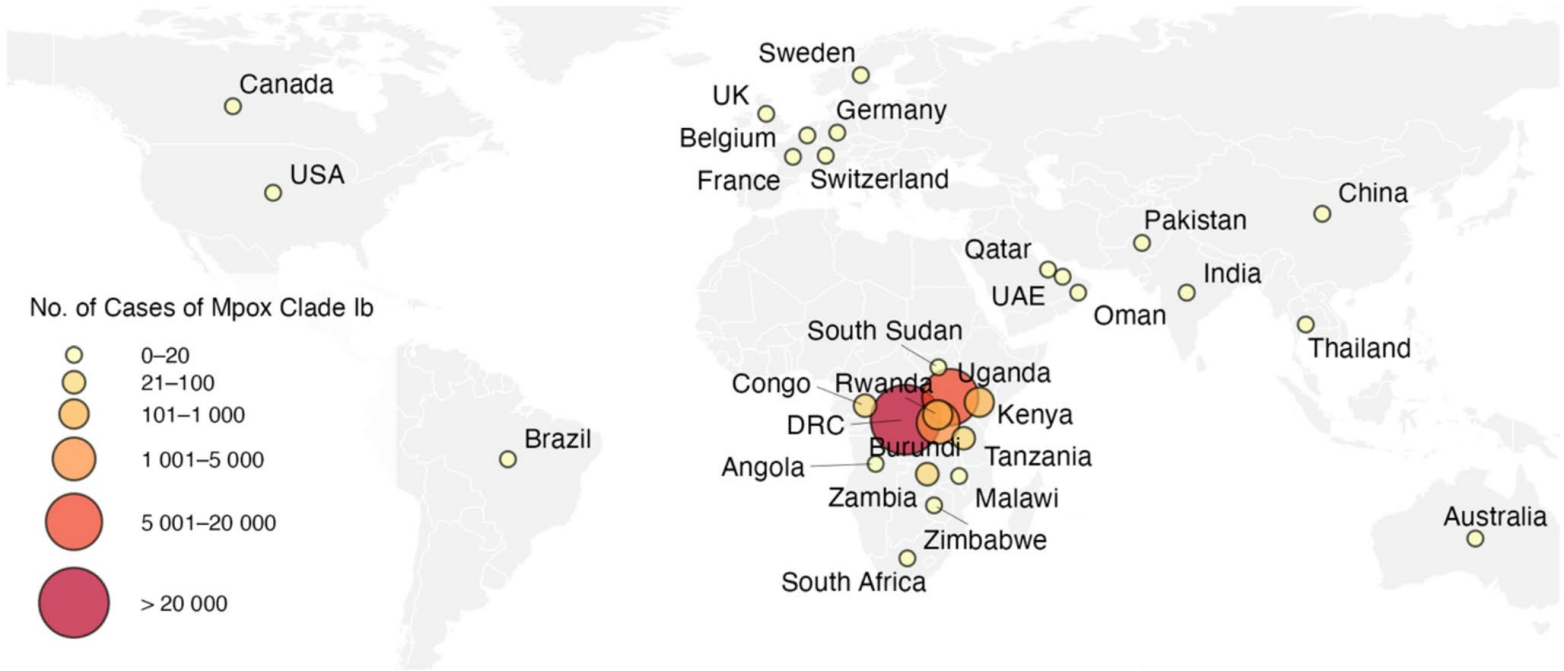
Geographic distribution of confirmed Clade Ib Mpox cases from 01 January 2024 to 02 June 2025. Circles indicate the number of confirmed cases of Mpox clade Ib per country, as reported to the World Health Organization. Circles are positioned at the geographic centroid of each country. As of 2 June 2025, a total of 33,889 confirmed cases of Mpox clade Ib were reported worldwide, across 30 countries, with the Democratic Republic of the Congo accounting for more than two-thirds of total cases (67.6%). Countries reporting community transmission of Mpox clade Ib are, in descending order of number of cases: the Democratic Republic of the Congo (*n* = 22,896), Uganda (*n* = 6479), Burundi (*n* = 3960), Kenya (*n* = 133), Rwanda (*n* = 120), Zambia (*n* = 82), the Republic of the Congo (*n* = 63), the United Republic of Tanzania (*n* = 47), South Sudan (*n* = 15), Malawi (*n* = 12), and South Africa (*n* = 6). The United Kingdom is classified as having travel-associated cases, except for one case reported with no travel history or links to infected travelers (i.e., community transmission). All cases reported outside the African continent (except one case in the United Kingdom) are linked to travel in African countries with community transmission of MPXV clade Ib. Countries reporting cases linked to travel are, in descending order of number of cases: the United Kingdom (*n* = 12), Germany (*n* = 10), China (*n* = 10), India (*n* = 10), South Africa (*n* = 6), Belgium (*n* = 5), Thailand (*n* = 5), Qatar (*n* = 5), the United States of America (*n* = 4), France (*n* = 3), Angola (*n* = 2), United Arab Emirates (*n* = 2), followed by Brazil, Switzerland, Canada, Oman, Pakistan, Sweden, Zimbabwe, and Australia, with a single case each. Note: In countries classified as having travel-associated cases, only cases associated with MPXV clade Ib are represented. Cases for which the clade and subclade have not been determined are not included. Cases reported in the DRC and the Republic of the Congo are known to be a mix of MPXV clade Ia and Ib. Data source: World Health Organization (https://worldhealthorg.shinyapps.io/mpx_global/#sec-down). Map generated using RStudio software (v2024.4.2.764). Abbreviations: USA, United States of America; UAE, United Arab Emirates; DRC, the Democratic Republic of the Congo

Sustained human-to-human transmission of MPXV clade Ib resulted in 2,672 confirmed cases in South Kivu between 1 January and 20 October 2024 [84]. Clade Ib MPXV subsequently spread to other provinces of the DRC and, from summer 2024, to neighboring African countries, including Burundi, Rwanda, and Uganda, followed by Kenya and Zambia. In response, the Africa Centers for Disease Control and Prevention and the WHO declared the mpox epidemic a PHEIC in August 2024 [16, 85]. Following the first reported case of mpox clade Ib in Sweden in mid-August 2024, additional cases were reported, notably in Thailand, India, Germany, the UK, the US, Canada, Pakistan, Belgium, China, South Africa, Angola, Brazil, France, and more recently Australia, for a total of 82 cases in countries without known community transmission of clade Ib as of 2 June 2025 [86] (Fig. 2). Most cases occurred in travelers returning from affected African countries, although limited secondary transmission have been reported in the UK, Northern Ireland, Germany, Belgium, China, Brazil, and France [87].

Beyond the recent emergence of clade Ib MPXV, the current spread of mpox across several African countries is also linked with the spread of clade Ia MPXV, especially in historically endemic provinces of the DRC [88]. Unlike clade Ib, clade Ia has been responsible for recurrent outbreaks over many years in central Africa, primarily related to repeated animal spillovers, leading to high genetic diversity and co-circulation of multiple lineages within and across provinces [89]. Clade Ia viral evolution largely occurred within its animal reservoir, resulting in low levels of APOBEC3-like mutations. Initially, the progressive rise in clade Ia cases was linked, as with other MPXV clades, to broader ecological and socio-political drivers — deforestation, urban expansion into rainforest areas, armed conflict, poverty, and forced population displacement — that increase human exposure to animal reservoirs and reduce outbreak containment capacity [90]. However, recent data suggest that human-to-human transmission has been sustained in Kinshasa since mid-2024, particularly through sexual contact. Similar patterns have subsequently been observed in other provinces [91, 92]. Clade Ia viruses associated with these outbreaks exhibit an APOBEC3-like mutational signature, raising concerns about enhanced viral adaptation and the potential for broader dissemination.

Africa is currently experiencing concurrent outbreaks of MPXV clades Ia, Ib, IIa, and IIb, with 26 countries reporting 32,906 confirmed cases over the last 12 months as of 1 June 2025, including 120 deaths. The DRC remains the most affected country, with 22,896 cases related to clades Ia or Ib reported between 1 January 2024 and 2 June 2025 [17, 88]. Notably, Sierra Leone has experienced a concerning surge in mpox cases caused by clade IIb, lineage A.1 - the same lineage implicated in the 2017 outbreak in Nigeria. While the historic burden of mpox was very low in Sierra Leone until Decembre 2024, the country reported a total of 3,782 confirmed cases between January and 31 May 2025 [88]. Young adults aged 16–35 years represent more than two-thirds of cases [93]. Alarmingly, Sierra Leone is now the third most affected country in Africa in terms of new mpox cases reported in 2025 - after the DRC and Uganda - and has become the epicenter of a westward expansion of mpox across the continent. The outbreak is now extending to neighboring countries, with Liberia reporting 113 new cases in 2025 to date. Cases reported in Sierra Leone over the past six weeks account for nearly half of all new mpox cases across Africa [88]. Emerging data suggest that this near-exponential growth is being driven by sustained human-to-human transmission, primarily via sexual contact [93].

## Clinical presentation

The clinical presentation of mpox cases during the 2022 outbreak differed significantly from previous epidemics and may have been influenced by the route of transmission involved, but also host-related factors. While the median incubation period in previous epidemics ranged from 4 to 21 days, it was shortened to 7–10 days after exposure during the 2022 outbreak [11, 34, 79, 94–96]. This shorter incubation may be related to direct viral inoculation during sexual intercourse, as already demonstrated in cases of invasive contact with infected animals (bites, scratches) compared with non-invasive contact in endemic areas [34].

After this asymptomatic incubation, mpox patients generally experienced systemic symptoms 1 to 5 days before skin rash onset, including myalgia, fatigue, headache, fever, and generalized lymphadenopathy, which constitute the prodromal phase [11, 34, 96–98]. During the 2022 outbreak, systemic symptoms were common (62–100% of patients) [11, 97, 98] and occurred sometimes simultaneously or shortly after the rash (early clinical course) [79]. Generalized lymphadenopathy was observed in only 30% of cases, compared with 49–60% during previous outbreaks, with lymphadenopathy being confined to the lymph catchment area of skin or mucosal lesions in 49–85% of patients [8, 24, 79, 99] (Table 1).

**Table 1:** Overview of Mpox major outbreaks: clinical and epidemiological key features

	Clade Ib	Clade IIb lineage B.1	Clade IIb lineage A.1	Previous outbreak
General features				
Key geographical distribution	Central Africa, mainly Democratic Republic of the Congo	Global outbreak involving at least 122 countries	West Africa, mainly Nigeria	Mainly Central Africa, sporadic clusters in West Africa
Spread	Sustained human-to-human transmission. Heterosexual network and household	Sustained human-to-human transmission Mainly driven by close physical contact between MSM	Concurrent primary zoonotic and human-to-human transmission	Mainly zoonotic spillover with limited human-to-human transmission in rural areas adjacent or within rainforest
Outbreak period	2023 – ongoing	2022-ongoing	2017–2019	1970–2017
Estimated number of cases	33,889*	> 100,000	183	12,000–15,000
Population features				
Mean age	22 years	34–46 years	29–30 years 80% of cases > 20 years	12.6–25 years 80% of cases < 15 years before 1997
Male	53%	88–99%	69-70.1%	53.8–62.1%
Living with HIV	2%	24–48%	27.9%	ND
Concomitant STI	ND	29–41%	ND	ND
History of STI ≤ 12 months	ND	25 − 50	ND	ND
Clinical features				
Incubation period	7.3–9.9 days	7–10 days	ND	4–21 days
Systemic symptoms	Fever (58%) headache (23–60%), fatigue (91%), myalgia (22–73%) Malaise (77%), sore throat (26%)	Fever (47–72%), headache (16–53%), fatigue (23–41%), myalgia (14–55%), malaise (57%), sore throat (24%)	Fever (88–90%) headache (62–79%), fatigue (52%), myalgia (23–63%), malaise (57–61%), sore throat (45–58%)	Fever (45–90%), myalgia (73–85%), headache (48%), sore throat (63%)
Lymphadenopathy	42–73%, mainly localized (64%)	Generalized (30%), confined to the lymph catchment area (49–85%)	62–69% localized or generalized	53–84% localized or generalized
Rash features				
Number of lesions	< 25 : 24% 25–99 : 42% 100–250 : 19% > 250 : 15% Median lesions count : 42 (17–94)	1–10 : 63–87% 11–20 : 21% > 20 : 8–11% ≥ 100 : 0–4%	< 100 : 40% 101–1000 : 42.5% ≥ 1000 : 17.5%	< 100 : 33.3% ≥ 100 : 66.7%
Area of distribution	Head and neck (72%), trunk (55%), arms (80%), legs (69%), genitals(79%), perianal (34%), palms (41%), soles (25%), oral (25%)	Face (20–39%), trunk (25–57%), limbs (50–60%), palm and soles (10–60%), genitals (46–61%), perianal (36–44%), oral (20–43%), perioral (28%)	Face (96–97%), legs (85–91%), trunk (80–92%), arms (79–87%), palms (55–69%), genitals (62.5%−68%), sole (50–64%), oral (37.5%)	Face (84%), palm (69%), genitals (67%), sole (58%), oral (50%)
Distribution	Disseminated to > 3 body areas in 35%, predominantly genital in 21%	Commonly limited to 1–3 body areas	Commonly disseminated to > 3 body areas	Commonly disseminated to > 3 body areas
Progression	ND	42% of cases with asynchronous progression of lesion, not all lesion progressing through the 4 stages in order	Progression through 4 stages in order	Progression through 4 stages in order
Outcomes				
Complications	Genital oedema (41%), urethritis (40%), secondary bacterial infection (19%), proctitis (5%), conjunctivitis (2.7%), encephalitis (0.7%),bronchopneumonia (0.6%),	Rectal pain (14–36%), proctitis (11–25%), penile oedema (8–16%), swallowing difficulty (5–14%), secondary bacterial infection (3–4%), conjunctivitis (1%)	Secondary bacterial skin infection (47.5%), sepsis (10%), bronchopneumonia (7.5%), encephalitis (7.5%), keratitis (7.5%)	Secondary bacterial skin infection (60.9%), bronchopneumonia (17.3%), keratitis (4.3%), sepsis (0.4%), encephalitis (0.4%)
Hospital admission rate	ND	1–13%	12.7%	23–61%**
Case-fatality rate	0.3–1.4%	< 0.1% 15% in people with advanced HIV	3.6%	Clade I :10.6% Clade IIa : 1%

Data were retrieved from the following references : [2–5, 7, 8, 10, 11, 63, 78, 79, 84, 88, 97, 98, 101, 126, 128, 129, 188–203]

*ND* no data; *MSM* men having sex with men; *STI* sexually transmitted infections

*As of 1 May 2025. The cases reported in the Democratic Republic of the Congo, which account for the vast majority, are known to be a mix of MPXV clades Ia and Ib

**Very limited evidence

The key clinical feature of mpox is diffuse, painful skin lesions evolving uniformly over a period of 2 to 4 weeks and progressing successively through 4 stages: macules, then papules, vesicles with solid content and finally pustules [100]. However, several cases in the 2022 outbreak exhibited asynchronous lesion progression [101]. The skin lesions are usually well circumscribed and present a central umbilication. At the end of the eruptive phase, lesions dry out, crust over and eventually fall off. The fall of the last lesion usually marks the end of the patient’s contagiousness [102].

The number of mpox skin lesions can vary widely, ranging from a few to several hundred. In immunocompromised individuals, such as those with advanced HIV infection (CD4 count < 200/mm^3^), profuse involvement with over 1,000 lesions has been reported [22, 103]. Lesions count also distinguishes the 2022 outbreak from earlier ones, with over two-thirds of patients presented with more than 100 lesions during local epidemics in endemic areas, compared with only 0–4% during the global 2022 outbreak [11, 22, 97, 100]. Up to 12% of patients during the 2022 outbreak presented with a single lesion, potentially leading to delayed or missed diagnosis in the context of sexual transmission [11, 79, 97]. However, single lesions without other symptoms only occurred in less than 5% of mpox patient during the 2022 outbreak. Single lesions at inoculation sites were also described in cases of percutaneously transmitted mpox (e.g., needle-stick injuries) [104].

**Fig. 3:**
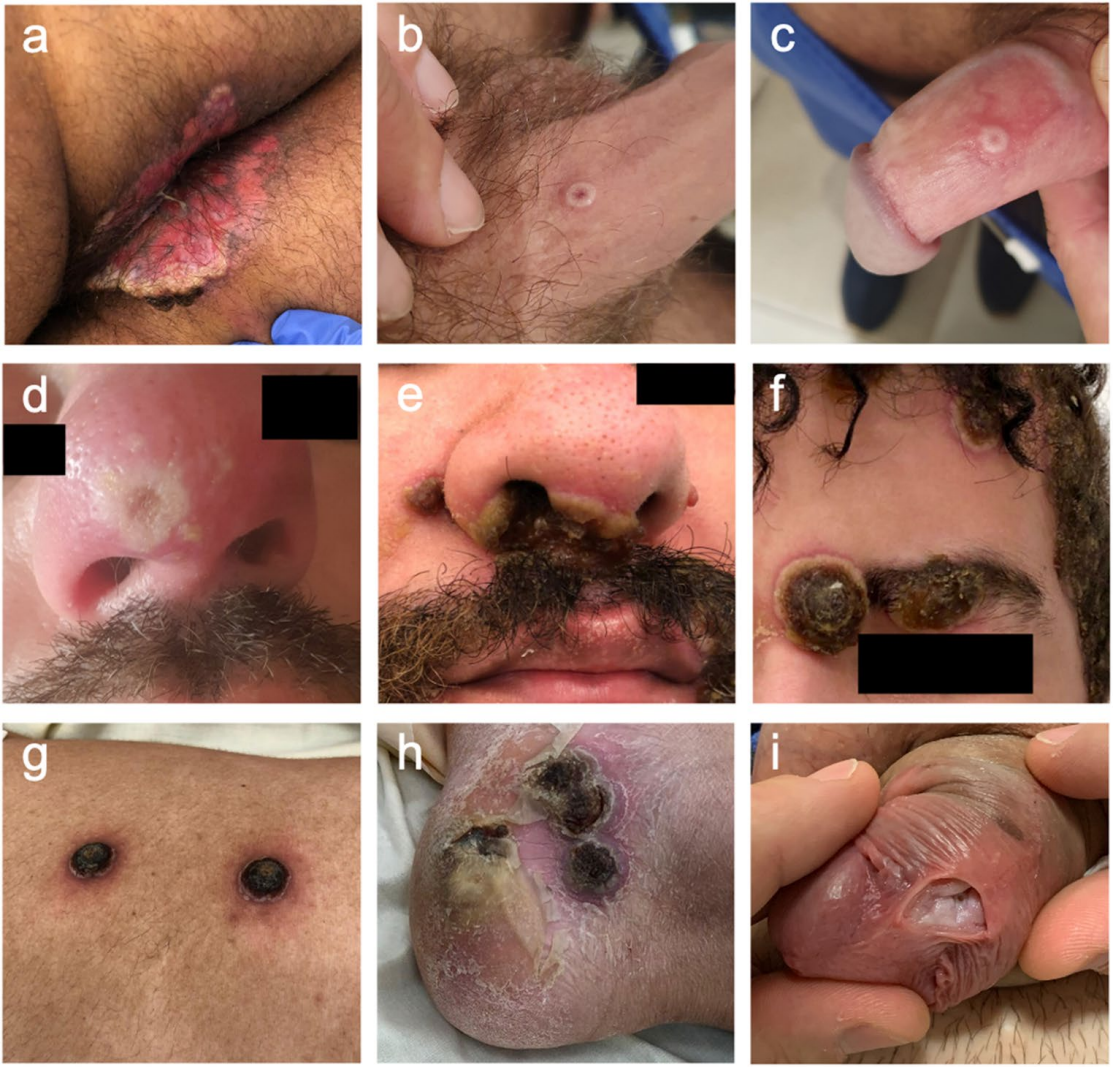
Cutaneous and mucosal manisfestations of mpox. Panel **a**: perianal rash with ulceration. Panels **b-d** : all photos are from a single patient, presenting with typical umbilicated pseudopustules on the penis (**b**, **c**) and a single umbilicated pseudopustule on the nose with peripheral oedema (**d**). Panels **e-h** : all photos are from a single patient with advanced HIV presenting with a severe and protracted course of mpox, with large crusted lesions on the face (**e**, **f**), trunk (**g**), and foot (**h**). Facial lesions had superinfection documented with *Klebsiella aerogenes* and *Staphylococcus lugdunensis.* Panel **i**: Cutaneous defect with a fibrous base, without evidence of urethral fistula. This sequela was observed four months after mpox infection, presenting as a single lesion on the posterior aspect of the penis in a patient with HIV-controlled infection Photo credits : Guillaume Martin-Blondel (**b-d**), Xavier Boumaza (**a, e-h**), Marie Piffaut (**i**)

During mpox outbreaks from 1970 to 2021, rashes mainly involved the trunk (80–100%), limbs (71–97%), and face (92–98%), and to lesser extent the genital (28–67%) and oral (24–55%) areas [8, 105–107]. Conversely, patients during the 2022 outbreak mainly presented with lesions in the genital (46–61%), perianal (36–44%), perioral, and oral areas (14–43%) with lesser involvement of the trunk (25–57%), limbs (50–60%), and face (20–39%), and no centrifugal distribution [11, 76, 79, 101] (Fig. 3). Mucosal lesions affected around 40% of patients, including proctitis, pharyngitis or urethritis. Anorectal symptoms (tenesmus, diarrhea, anorectal pain) and oropharyngeal symptoms (odynophagia, epiglottitis, oral or tonsillar lesions) were the presenting symptom in 12% and 5% of patients, respectively [11]. This distribution pattern suggests a link between the site of inoculation and the lesion location; the 2022 epidemic being primarily driven by a sexual route of transmission. Data from a cohort of 181 individuals further support this hypothesis: MSM were more likely to develop anorectal and pharyngeal lesions following receptive anal or oral sex, respectively, as well as a quantitative association between penile, anorectal, and oral exposure and same-site lesion development, as observed in a GeoSentinel cross-sectional study of 226 mpox patients [79, 108].

## Complications and sequalae

Historically, severe complications of mpox have included ocular involvement, secondary bacterial infections, respiratory, and neurological manifestations.

Ocular involvement includes conjunctivitis, corneal ulcers, keratitis, lesions of the eyelids or periorbital cellulitis, with a risk of blindness due to corneal scarring [109]. Respiratory complications, reported in up to 12% of mpox patients, are associated on the one hand with direct MPXV-related lung injury, as demonstrated in non-human primate models developing focal pulmonary necrosis, multifocal nodular pneumonia, diffuse pulmonary consolidation and fulminant bronchopneumopathy after MPXV exposure, and on the other hand with viral co-infections or bacterial superinfections [24, 110]. Data gathered in the US during the 2022 outbreak suggest that respiratory complications may be more common in people living with HIV [111]. Mpox neurological manifestations included encephalitis (2%), seizures (2.7%), confusion (2.4%), as well as myalgia, fatigue, and headache in a systematic review of mpox studies before the 2022 outbreak [112]. Skin complications are relatively common, including necrotizing lesions with bacterial superinfection, diffuse maculopapular rashes and ulcerations, especially in individuals with advanced HIV infection and children [113]. Mpox can also lead to skin sequelae, including pockmarks, pitted scars, and patchy alopecia [114].

The 2022 outbreak was associated with a range of novel severe complications, including sporadic cases of myopericarditis, epiglottitis, paraphimosis, peritonsillar abscess, rectal wall perforation, and hemophagocytic lymphohistiocytosis [97, 115–118]. Other less severe but more common complications included penile edema (8–16%), proctitis (11–25%), impaired swallowing (5–14%), and secondary bacterial infections (3–4%) [119].

Bacterial superinfections remain the most frequent complication in pediatric cases, accounting for up to 60% of complications according to the meta-analysis by Sanchez Clemente et al. [120].

## Prognosis

In most patients, mpox is a self-limiting disease with moderate symptoms resolving spontaneously within 2 to 4 weeks and typically does not require inpatient care. Conversely, certain groups are considered at high risk of progression to severe disease, including children, pregnant women and immunocompromised individuals, such as those with advanced or uncontrolled HIV infection and solid organ transplant recipients [121].

Severe cases involving pregnant women and children were commonly reported during earlier epidemics caused by clade I MPXV, but only a few were described during the clade IIb outbreak in 2022 [24, 96, 120, 122–124]. Compared to healthy adults, children are at higher risk of severity and hospitalization, with a pooled Case Fatality Rate (CFR) calculated at 11% (95% CI 4–20). The burden of severe pediatric cases is largely driven by endemic African countries, where structural vulnerabilities such as malnutrition and limited access to healthcare may further increase clinical severity. Although data on mpox infection during pregnancy remain scarce, pregnant women are also considered at high risk of severe disease, including a high rate of fetal loss – reported in up to half of infections acquired during pregnancy regardless of the trimester – and a risk of congenital mpox [120].

In people living with HIV, the risk of severe and protracted forms of mpox appears to depend on immunological status [113]. Indeed, individuals with uncontrolled or advanced HIV infection were at higher risk of severe or prolonged form of mpox during the 2017–2018 epidemic in Nigeria, with 4 of the 7 deceased patients having acquired immunodeficiency syndrome (AIDS) in a cohort of 122 cases [8]. Similar findings were reported during the 2022 outbreak in a cohort of 57 US patients hospitalized for severe mpox, 82% of whom were HIV-positive, with 90% having CD4 counts < 200 cells/mm^3^ [125]. In contrast, reports from the 2022 outbreak did not show increased risk of severe disease, hospitalization, or death in people living with HIV receiving effective antiretroviral therapy [11, 79, 97]. Interestingly, 2022 data from the GeoSentinel Network revealed that patients with well-controlled HIV infection were nonetheless at increased risk of developing a higher rash burden compared to people who are not infected with HIV [101].

Hospitalization rates during the 2022 outbreak ranged from 1 to 13%, depending on the study, much lower than rates reported during previous outbreaks in endemic regions, which reached up to 26% [119]. Hospitalization was primarily intended for isolation, pain management, or management of complications, and to a lesser extent, for specific antiviral therapy [126, 127].

The CFR associated with MPXV infection has historically been higher for the Central African clade I than for the West African clade II, with a pooled CFR estimate of 10.6% (95% CI: 8.4–13.3%) for clade I versus 3.6% (95% CI: 1.7–6.8%) for clade II, according to a meta-analysis by Bunge et al. [5]. Notably, the 2017–2018 outbreak in Nigeria (clade IIb lineage A.1) accounted for most clade II-related deaths in that study, while no deaths were reported in other West African countries (Côte d’Ivoire, Liberia, and Sierra Leone) or among cases reported outside Africa (USA, UK, Singapore, Israel). The CFR associated with the 2022 outbreak linked to clade IIb lineage B. 1 were even lower, estimated at less than 0.1%, with most deaths occurring in immunocompromised individuals with advanced HIV infection (CFR: 15%, CD4 counts < 200 cells/mm^3^) [113, 128]. More recently, the subclade Ib outbreak that emerged in the DRC in September 2023 appeared to be associated with a lower CFR than previous clade Ia outbreaks. Among hospitalized patients, estimated CFRs ranged from 0.5 to 1.4%, while syndromic surveillance data suggest an overall CFR of 0.3% (95% CI: 0.2–0.4%) in South and North Kivu [63, 84, 88, 129].

Such differences in clinical severity could be, at least partially, explained by genetic variation across the different MPXV clades and subclades, occurring in regions encoding for important virulence genes. Epidemiological factors may also explain differences between outbreaks, such as a higher proportion of infected children, known to be at risk of severe disease, during previous clade I outbreaks in African countries where transmission occurred primarily through household contact, whereas the 2022 global outbreak mainly affected adult MSM. In addition, disparities in access to supportive care and nutrition may account for differences observed between high-income and low- and middle-income countries. The PALM007 study, conducted between 2022 and 2024 in children and adults infected with clade I MPXV in the DRC, showed a mortality rate of 1.67% in the context of high-quality supportive care, much lower than the CFR of 4.6% observed under real-world conditions in the DRC in 2023 [130]. To note, CFR estimates should be interpreted with caution, as they are subject to measurement bias, particularly in settings with limited diagnostic capacity which may result in underreporting of mild or asymptomatic cases, and consequently lead to an overestimation of the true CFR.

## Diagnosis

Mpox diagnosis is suspected on known or possible exposure and clinical findings, as detailed above in the Clinical Presentation section. A combination of clinical suspicion and epidemiological risk factors for mpox infection defines the case as probable [131, 132].

The diagnostic approach depends on the resources allocated to it. When resources permit, current recommendations are to test all individuals meeting the definition of a suspected or probable case of mpox infection. In resource-limited settings, biological testing should be prioritized for specific patient groups, such as young children (particularly those under five years of age), individuals presenting with severe and/or atypical clinical manifestations, and those at risk of severe disease (e.g., immunocompromised individuals). Testing should also be performed in symptomatic individuals originating from non-endemic areas, those without known epidemiological link to confirmed cases, and healthcare workers [133].

Diagnostic confirmation is achieved through nucleic acid amplification testing (NAAT) on lesions samples, primarily cutaneous, using either a swab of the lesion or lesion contents, or a lesion crust. To ensure adequate viral DNA collection, swabbing must be carried out vigorously, without prior unroofing or aspiration of lesions [134]. At least two lesions from two different sites should be swabbed. For sample collection, the European Centre for Disease Prevention and Control (ECDC) recommends that clinicians wear single-use gloves, a gown, eye protection, and an FFP2 mask [135]. Swabs should be transported to the laboratory either dry or in a viral transport medium, according to local laboratory recommendations.

NAAT testing on non-skin samples (e.g., oral, nasopharyngeal, or anorectal) is not currently recommended as routine practice, but may be useful in cases with sole mucosal involvement, or for epidemiological and research purposes. Positive NAAT results in serum have also been reported, but their clinical significance remains unclear [39, 136].

Point-of-care diagnostic testing is essential for controlling the spread of mpox. Rapid identification of infected individuals using highly sensitive and specific assays is crucial. This enables prompt implementation of effective isolation measures, initiation of appropriate antiviral therapy, and systematic identification and monitoring of high-risk contacts. In addition to molecular methods for direct pathogen detection, rapid antigen tests are available and can be performed on skin lesion samples or oropharyngeal swabs. However, due to their currently limited analytical performance, these tests are not recommended [133, 137].

Serological tests have limited value in mpox diagnosis due to immunological cross-reactivity among human-pathogenic orthopoxviruses. However, they may be useful for ruling out a recent or past diagnosis of orthopoxvirus infections or for conducting population-based serosurveys. For these purposes, Immunoglobulin M (IgM) and Immunoglobulin G (IgG) detection by enzyme-linked immunosorbent assay (ELISA) or immunofluorescent antibody assay (IFA) is available in some laboratories [138].

Skin biopsy is rarely used for mpox diagnosis given the similarity of its histological features with other human orthopoxvirus infections.

Finally, the ECDC recommends that all diagnostic procedures for MPXV and specimen handling be performed in biosafety level-2 facilities as a minimum requirement. Handling of mpox-positive samples may be subject to strict legal restrictions in some countries, requiring specific authorization from national authorities [138].

## Differential diagnosis

Several differential diagnoses should be considered in cases of mpox, including other poxvirus infections, other common viral infections, STIs, other common bacterial skin infections, and various non-infectious skin conditions. The key characteristics distinguishing mpox from these differential diagnoses are presented in Table 2.

**Table 2:** Differential diagnosis of dermatological manifestations of Mpox

Differential diagnosis	Key differential features with mpox
Chickenpox	• Lymphadenopathy is usually absent • Asynchronous shallow vesicles • Centripetal extension • Usually respect the palms and soles
Herpes simplex infection	• Non-deep-seated vesicles • History of recurrent localized rash
Herpes zoster	• Unilateral lesions with dermatomal distribution • Intense pain precedes the rash
Measles	• Blotchy red rash with cephalocaudal spread • Absence of vesicular lesions • Koplik spots
Molluscum contagiosum	• Flesh-colored localized papular umbilicated lesion, without vesicular stage
Tanapox	• Few localized nodular lesions • Usually ulcerate without becoming pustular
Orf	• Contact with livestock • Usually solitary lesions, without systemic illness
Borealpox	• Exposure in subarctic North America
Smallpox	• Clinical distinction between smallpox and mpox is challenging; however, smallpox remains an exceedingly unlikely diagnosis given its eradication in 1980 • Lymphadenopathy is usually absent
Hand-foot-and-mouth disease	• Lymphadenopathy is usually absent • Shorter rash duration (< 10 days) • Rash distribution: oral cavity, palms and soles
Secondary syphilis	• Absence of vesicular lesions • Painless lesions
Disseminated gonococcal infection	• Erythematous macules developing into vesiculopustular, or necrotic papules, or hemorrhagic bullae • Fewer lesions (< 30) on the extremities • Rheumatic symptoms • Lymphadenopathy is usually absent
Chancroid	• Painful genital ulcer with unilateral suppurative lymphadenopathy, buboes in later stages • Typically no vesicles or pseudopustules
Lymphogranuloma venereum (LGV)	• Small genital/rectal ulcers associated with tender inguinal or femoral lymphadenopathy (“groove sign of Greenblatt”)
Eczema vaccinatum	• History of atopic dermatitis and recent smallpox vaccination
Eczema herpeticum	• History of atopic dermatitis • Innumerable small papules evolving into fragile vesicles then crusting
Impetigo	• Superficial pustules with honey-colored crust • No systemic illness
Drug reaction	• Polymorphic generalized rash • History of recent new drug exposure
Erythema multiforme	• Target lesions • Usually symmetric acral distribution
Bullous pemphigoid	• Large tense bullae, on erythematous base with symmetrical distribution • Usually in elderly patients
Dermatitis herpetiformis	• History of celiac disease • Grouped vesicles and excoriated papules, intensely pruritic, symmetrically distributed on extensor surfaces

Chickenpox is the primary differential diagnosis in mpox patients presenting with skin lesions. However, several simple clinical features differentiate the two conditions. Chickenpox typically presents with skin lesions at different stages and fluid-filled vesicles, whereas mpox lesions tend to be more synchronous and contain solid material (pseudopustule). The distribution of lesions respecting the palms and soles and the absence of lymphadenopathy also distinguish chickenpox from mpox.

Other poxviruses should be considered, including Molluscum contagiosum, which presents with multiple papules featuring central umbilication; Tanapox virus causing rashes lasting several weeks and preceded by a febrile prodromal phase, mainly described in African countries; or Borealpox presenting with localized skin lesions and regional lymphadenopathy after exposure in subarctic North America. Orf, a parapoxvirus infection, can cause skin lesions relatively similar to those of mpox. However, exposure at risk differs, as Orf is typically associated with contact with livestock [139].

Several STIs must be considered in the differential diagnosis, including primary syphilis presenting with perineal ulcerations, lymphogranuloma venereum, gonorrhea, or syphilis in patients with proctitis; and herpes simplex virus infection in those presenting with perioral vesicles or anal ulcers. Beyond being a differential diagnosis, concomitant active STIs were found in 29–41% of mpox patients during the 2022 outbreak, reinforcing the need to offer comprehensive STI testing in patients diagnosed with mpox [11, 77–79].

Finally, other bacterial skin infections such as impetigo caused by group A *Streptococcus* or *Staphylococcus* spp. should be considered, as well as non-infectious conditions such as dermatitis herpetiformis [140].

## Treatment

### Supportive care

Mpox is typically a self-limiting and mild disease; therefore its management is primarily based on symptomatic and supportive care, usually carried out outside of the hospital (87% outpatient management during the 2022 outbreak in the Thornhill et al. cohort) [11].

The Centers for Disease Control and Prevention (CDC) provides guidance on pain management [141]. It relies on oral systemic pain medication as well as topical agents (e.g., local anesthetics). In cases of severe or refractory pain, gabapentin or opioids may be necessary. For proctitis, laxatives and lidocaine gels may be helpful. In cases of suspected or documented bacterial superinfection, topical or systemic antibiotic therapy is recommended. Intravenous therapy may be required in the most severe cases, particularly when oral mucosal damage impar the intake of treatment, food or fluids.

### Medical treatment

There is no treatment specifically developed for mpox to date. Currently, four antiviral agents are available, namely tecovirimat (intravenous and oral), cidofovir (intravenous and topical), brincidofovir (oral) and trifluridine (topical); as well as vaccinia immune globulin intraveinous treatment (VIGIV) [142] (Table 3). Among these, only tecovirimat has been granted marketing authorization for mpox treatment in the European Union (EU) by the European Medicines Agency (EMA), and was made available under a compassionate-use program during the 2022 outbreak [143]. Tecovirimat is not Food and Drug Administration (FDA) approved for mpox treatment, but was made available for selected patients in the US under the CDC-held Expanded Access-Investigational New Drug (EA-IND) protocol and through the STOMP trial during the 2022 global outbreak [144].

**Table 3:** Potential treatment options for Mpox

	**Tecovir imat**		**Cidofovir**	**Brincidofovir**	**Trifluridine**	**VIGIV**
Brand name	Tpoxx^R^		Vistide^R^	Tembexa^R^	Viroptic^R^	CNJ-016
**Route of administration**	PO	IV	IV	PO	Topical	IV
**Adult dosing**	40–120 kg : 600 mg x 2/day > 120 kg : 600 mg x 3/day	35–120 kg : 200 mg x 2/day > 120 kg : 300 mg x 2/day	5 mg/kg/week	200 mg/week	One drop/eye, every 2 h for 2 weeks then x4/day for 2 weeks. Max 9 drops/eye/day	6000–9000 IU/kg
**Renal dose adjustment**	None	Risk of hydroxypropyl-β-cyclodextrin accumulation (excipient). CrCl 30–89 ml/min) : none Severe (CrCl < 30 ml/min or dialysis) : contraindicated	Change in renal function during treatment : reduce to 3 mg/kg if increase in serum creatinine of 0.3–0.4 mg/dl above baseline. Preexisting renal impairment : CrCl ≤ 55 ml/min or proteinuria ≥ 100 mg/dl : contraindicated	None	None	Use VIGIV with caution in patients with pre-existing or at risk of renal impairment.
**Hepatic dose adjustment**	None		Safety and efficacy not established if hepatic disease	None Hepatic laboratory testing should be performed before and while receiving Brincidofovir.	None	None
**Administration**	Take within 30 min after a full meal containing moderate to high fat	6-h infusion. Dissolve in a 1:2 ratio.	1 h-infusion. IV prehydratation and Probenecid before any administration.	Take on an empty stomach or with a low-fat meal.	N/A	Rate of infusion : ≥ 50 kg : ≤ 2 ml/min. < 50 kg, ≤ 0.04 ml/Kg/min
**Duration of treatment**	14 days*		14 days*	14 days*	4 weeks	Single dose*
**Pregnancy, breastfeeding and fertility**	Likely safe		No recommended during pregnancy and breastfeeding. Might cause irreversible male infertility Women of childbearing potential and men should use effective contraception during treatment and at least 2 months after the last dose.		Likely safe with negligeable systemic absorption. Lack of experience in pregnant or breastfeeding women. Use only if benefits outweigh risks.	Likely safe
**Potential adverse drug reaction**	Headache, nausea, diarrhea, itching, abdominal pain.		Nephrotoxicity, neutropenia, acidosis, diarrhea, vomiting	Nausea, diarrhea, abdominal pain, nausea. Elevated liver enzymes. Potential human carcinogen	Adverse events associated with topical use (e.g. burning, stinging, eyelid oedema). Corneal epithelial toxicity if > 4 weeks	Anaphylaxis, volume overload, thrombosis, headache, acute kidney injury, other adverse event associated with immunoglobulin infusions.
**Drug interaction**	Rilpivirine : decrease Repaglinide : increase Midazolam : decrease		Nephrotoxic agents Many interactions related to probenecid	OATP1B1 and 1B3 inhibitors increase Brincidofovir exposition (e.g. protease inhibitors, fostemsavir, cobicistat)	No major interaction reported.	Vaccination with live-virus-vaccine should be differed for 3–8 months.
**FDA approval**	Smallpox		CMV retinitis in AIDS patients	Smallpox	HSV1 and HSV2-related primary keratoconjunctivitis and recurrent epithelial keratitis.	Complications of smallpox vaccination
**EMA approval**	Smallpox, smallpox vaccination complications, Cowpox, Mpox		CMV retinitis in AIDS patients – withdrawal of the marketing authorization in 2014	Orphan designation for smallpox, CMV prevention, adenovirus treatment in immunocompromised patients	Not approved	Not approved
**US availability**	CDC EA-IND		Commercially available	FDA e-IND	Commercially available	CDC EA-IND
**EU availability**	Commercially available		Cidofovir tillomed pharma available in Germany, Spain, Belgium.	No	No	No
**Main trial for Mpox**	PALM007 - NCT05559099 STOMP - NCT05534984 UNITY - NCT05597735 EPOXI - NCT06156566		Limited to case reports and preclinical studies	MOSA - NCT0114318	Limited to case reports and preclinical studies	Limited to case reports and preclinical studies

Abbreviations: *VIGIV* Vaccinia Immune Globulin Intravenous; *PO* Per Os; *IV* intravenous; *IU* international unit; *CrCl* creatinin clearance; *N/A* non-applicable; *CMV* cytomegalovirus; *AIDS* Acquired Immunodeficiency Syndrome; *HSV* Herpes Simplex Virus; *FDA* Food and Drug Administration; *EMA* European Medicines Agency; *CDC* Centers for Disease Control and Prevention; *EA-IND* Expanded Access Investigational New Drug; *e-IND* Electronic Investigational New Drug; *EU* European Union

*May be extended in severe infection or immunocompromised patients

So far, there is no evidence-based effective antiviral therapy for mpox, and all randomized controlled trials conducted to date have shown inconclusive results regarding clinical efficacy.

### Tecovirimat

Tecovirimat (Tpoxx^R^) is considered the first-line treatment, according to European and American guidelines, for patients with severe mpox patients or for those at risk of developing a severe form of the disease (including children under 8 years of age, severely immunocompromised patients such as those living with HIV with CD4 counts < 200/mm^3^ or solid organ transplant recipients, pregnant or breastfeeding women and atopic dermatitis patients) [138, 144]. Tecovirimat is a virostatic antiviral that targets the orthopoxvirus p37 protein, thereby blocking cell-to-cell viral transmission. Tecovirimat’s efficacy in treating smallpox was established based on preclinical studies, including four studies in lethal non-human primate models, complemented by safety data from 359 healthy adults, leading to its approval by the FDA in 2018, followed by the EMA and the UK for the treatment of smallpox [143, 145, 146]. The EMA and the UK subsequently extended its approval to mpox, cowpox and complications related to smallpox vaccination in January 2022 [143, 147]. At the time of the 2022 global outbreak, evidence of the efficacy of tecovirimat in mpox treatment in humans was limited to a few observational studies, reporting anecdotal improvements in symptoms and viral clearance [148–150]. However, tecovirimat has been shown to be active in vitro against the MPXV IIb lineage B.1 [151]. Since then, the efficacy of tecovirimat has been evaluated by the STOMP study, an international randomized, placebo-controlled trial, involving participants non-severely ill with clade IIb mpox for less than 14 days. The trial began in September 2022. Non-pregnant, non-breastfeeding, non-severely immunocompromised adult participants were randomized at a two-to-one ratio to receive tecovirimat or placebo, while children, pregnant women and severely immunocompromised subjects were assigned to an open-label study arm (i.e. all receiving tecovirimat). In December 2024, a planned interim analysis showed that tecovirimat was not effective in reducing the time to lesion resolution and decreasing pain. Given the design of the study, no conclusion could be drawn regarding subjects with severe mpox or at risk of developing severe clade II mpox [152]. These results are consistent with those of the PALM007 study released in April 2025, a placebo-controlled trial involving 597 children and adults (including pregnant women) infected with clade I MPXV in the DRC, randomly assigned in a 1:1 ratio to receive tecovirimat or placebo. The PALM007 trial showed no significant differences between placebo and tecovirimat with regard to the time to lesion resolution or declines in the proportion of positive PCR results [130]. However, the overall CFR in the placebo group was lower than expected, suggesting that hospitalization and supportive care can improve outcomes. No safety concerns were observed with tecovirimat use in either PALM007 or STOMP trials. As of 21 April 2025, the efficacy and safety of tecovirimat are still being evaluated in two international randomized controlled trials: the UNITY trial (NCT05597735), in which nearly half of enrolled participants to date are HIV-positive, and the European EPOXI trial (NCT06156566). Of note, several cases of phenotypic resistance have been reported, mainly in severely immunocompromised patients receiving prolonged courses of tecovirimat. Transmission of resistance has also been observed in the US [153].

#### Cidofovir and brincidofovir

Cidofovir inhibits viral DNA polymerase and is approved by both the EMA and FDA solely for the treatment of Cytomegalovirus (CMV) retinitis in AIDS patients. However, cidofovir has demonstrated in vitro activity against MPXV and efficacy in lethal mpox animal models [154, 155]. Although no randomized efficacy data are available in humans, some reports suggest that cidofovir may be effective when administered intravenously, topically or via intralesional injection in severely immunocompromised patients [156, 157]. However, this theoretical efficacy must be weighed against the risk of dose-dependent nephrotoxicity, which can be reduced by coadministration with probenecid, and its embryotoxicity observed in animal models [158]. Brincidofovir, an oral prodrug of cidofovir, is considered less nephrotoxic than cidofovir. However, safety data from clinical trials on CMV infections in hematopoietic stem cell transplant patients indicate a risk of digestive (diarrhea) and hepatic toxicity, which may limit its use [159]. Brincidofovir has demonstrated efficacy against MPXV in animal models, and potential in vitro synergy with tecovirimat [160, 161]. It is currently FDA-approved for smallpox and can be used for mpox treatment in the US under an expanded access protocol. The CDC recommends its use in cases of severe mpox, either as monotherapy when tecovirimat is contraindicated or ineffective, or in combination with tecovirimat in severely immunocompromised patients [142]. Brincidofovir is not currently approved in the EU and the UK for mpox.

#### Trifluridine

Trifluridine is a topical antiviral agent conventionally used to treat Herpes Simplex keratitis and ocular vaccinia following autoinoculation. Although observational data on its efficacy are limited, the CDC recommends its use for mpox-related conjunctivitis and keratitis [142, 162**]**.

#### Vaccinia immune globulin intravenous

VIGIV, derived from sera of individuals recently vaccinated with a live vaccinia vaccine, is licensed only in the US for the treatment of complications of smallpox vaccination but was made available for mpox treatment during the 2022 outbreak under an EA-IND protocol [163]. There are currently very limited data supporting its efficacy in mpox patients [164]. Assuming that VIGIV could provide passive immunity against mpox through serological cross-reactivity among orthopoxviruses, the CDC recommends its use, (1) as a curative treatment for severe mpox in patients unable to mount a robust humoral response (e.g., severely immunocompromised patients); and (2) as a post-exposure prophylaxis in severely immunocompromised individuals or those contraindicated to vaccination [142].

## Prevention (behavioral, vaccination and hygiene strategies)

Preventing the spread of MPXV in both community and healthcare settings relies on a global strategy combining behavioral changes, vaccination (pre- and post-exposure), appropriate isolation, and hygiene measures.

### Vaccination

Smallpox vaccination using vaccinia virus is known to confer protection against mpox, as shown by a study conducted in Zaire in 1986, in which human-to-human transmission was five-fold lower in vaccinated individuals (0.9%) than in unvaccinated ones (7.2%) [165]. Although smallpox vaccination coverage ranged from 7 to 60% globally in 1980, routine vaccination has not been performed for more than 40 years following the eradication of smallpox in 1980 [166]. As a result, this cross-immunity currently benefits only people over the age of 40, whereas the mean age of cases in the 2022 global outbreak range from 34 to 46 years. Moreover, during previous outbreaks in endemic areas, most cases were under 40 years of age, with only 11–20% having received smallpox vaccination during childhood [119].

Four vaccines are currently licensed for mpox : (1) Modified Vaccinia Ankara-Bavarian Nordic (MVA-BN, also known as JYNNEOS^R^, IMVAMUNE^R^ and IMVANEX^R^), a third-generation live, attenuated, replication-deficient vaccinia vaccine, administered in two doses four weeks apart, licensed in the US, Canada, the UK, Nigeria, the DRC, Switzerland and the EU; (2) ACAM2000^R^, a single-dose, second generation, live, attenuated, replication-competent vaccinia vaccine, licensed in the US, Canada, Singapore and Australia [167]; (3) LC16m8, a third-generation, single-dose, highly attenuated vaccinia vaccine, only licensed in Japan and the DRC for mpox [168]; and (4) OrthopoxVac, a fourth-generation, live attenuated vaccinia vaccine licensed only in the Russian Federation.

While the ACAM2000 ^R^ vaccine has demonstrated efficacy against mpox in several non-human primate models, it may also cause moderate to severe adverse events related to replication-competent nature, including progressive vaccinia, eczema vaccinatum, generalized vaccinia, encephalitis, myocarditis, and pericarditis, thereby contraindicating its use in immunocompromised individuals, those with skin disorders (e.g., atopic dermatitis) or cardiac disease, or pregnancy [169].

Vaccination against mpox can be carried out as either pre- or post-exposure prophylaxis.

Preclinical studies of the MVA-BN vaccine in non-human primate models have demonstrated both immunogenicity and clinical efficacy against mpox [170]. These findings were later supported by several observational studies of MVA-BN administered as pre-exposure prophylaxis during the 2022 outbreak, which were collated in a meta-analysis by Pischel et al., concluding that the vaccine effectiveness (VE) of one dose and two doses MVA-BN was 76% (95%CI 64–88%) and 82% (95%CI 72–92%), respectively [171]. However, recent findings have raised concerns about the durability of protection conferred by the two-dose MVA-BN regimen. In individuals without prior childhood smallpox vaccination, neutralizing antibodies (NAb) titers have been shown to decline to baseline levels within 5–7 months post-vaccination, with only 3% of individuals retaining detectable NAb at 8-month follow-up. By contrast, 56% of individuals with prior smallpox vaccination retained detectable NAb over the same period [172, 173]. In this context, administration of an MVA-BN booster dose two years after the primary series in smallpox-naïve individuals was found to induce a rapid and robust increase in NAb, sustained at 6-month follow-up, comparable to the response observed in previously smallpox-vaccinated individuals following a single MVA-BN dose [173]. Recent data also suggest that similar immunogenicity can be achieved with a booster dose administered up to five years after the last dose [174]. Nonetheless, the long-term durability of this response beyond six months remains unknown and is currently being evaluated in the M-BOOST-FR trial (NCT06885853) over a 24-month follow-up period. Despite these findings, recommendations differ regarding the need for a booster dose in subjects at risk of non-occupational MPXV exposure. To date, only the French health authority recommends a single booster dose 24 months after the last dose for individuals fully-vaccinated during the 2022 outbreak, excluding immunocompetent individuals with prior smallpox vaccination [175]. For people at occupational risk of exposure to MPXV, e.g., lab workers, pre-exposure vaccination is recommended and booster doses are recommended (every 2 years for JYNNEOS^R^, every 3 years for ACAM2000^R^) [176]. Vaccination is not routinely recommended for clinicians with access to adequate personal protective equipment (PPE).

As expected given its attenuated phenotype, the MVA-BN vaccine appears to be safe, with a reported rate of myocarditis or pericarditis < 1 per 100,000 doses administered according to a real-world study based on Bavarian Nordic’s global safety database, and no such events were reported in phase II and III trials (including people living with HIV and those with atopic dermatitis). As a result, this third-generation vaccine is now approved for use in immunocompromised adults and adolescents. Its safety and immunogenicity in pregnant women are currently being assessed in the phase III PregInPoxVac trial (NCT06844500), and in children aged 2 to 12 years in a separate trial (NCT06549530), both conducted in the DRC, with enrolment starting in early 2025 and October 2024, respectively. The use of the MVA-BN vaccine has been limited by supply shortage and its high costs. To address this, several countries, including the US and several EU member states, have now authorized its intradermal administration, requiring only one-fifth (0.1 ml) of the volume used for subcutaneous injection (0.5 ml). Intradermal administration was initially adopted based on data from a phase II study demonstrating comparable antibody responses with both administration routes, and was further supported by cohort data indicating higher MPXV-specific IgG titers and a trend toward increased neutralizing antibodies, with comparable T-cell responses - albeit at the cost of slightly increased local reactogenicity [177, 178]. In addition, in September 2024, the WHO announced that MVA-BN would be the first vaccine against mpox to be added to its prequalification lists, to facilitate its acquisition by governments and international agencies, thereby improving access in low- and middle-income countries [179].

For pre-exposure prophylaxis, the CDC recommends, as of 21 April 2025, vaccination with JYNNEOS^R^ (two doses four weeks apart, subcutaneously or intradermally (in individuals over 18 years of age)) in individuals at risk of mpox. According to the CDC, individuals at risk of mpox include MSM, transgender or non-binary people, who within the last 6 months have had either a new diagnosis of ≥ 1 sexually transmitted disease, or ≥ 1 sexual partner, or sexual intercourse at a commercial sex venue or in association with a large public event in a geographic area where mpox transmission is occurring. This also includes sexual partners of individuals meeting the above criteria, or those who anticipate engaging in similar behaviors [180].

In response to the current clade Ib outbreak in Africa, the CDC now recommends that people travelling to countries with ongoing human-to-human clade I MPXV transmission should start vaccination at least six weeks before departure [181]. However, it should be noted that clinical data on virological efficacy against clade I viruses are currently lacking.

Vaccination against Mpox can also be used as post-exposure prophylaxis (PEP) in individuals with known or presumed exposure to MPXV. Vaccination should be carried out as early as possible, ideally within four days of exposure, although this period may be extended up to 14 days [176]. Beyond 14 days, vaccination should be considered on a case-by-case basis, weighing potential benefits and risks (e.g. severely immunocompromised individuals). However, vaccination is not recommended after symptom onset or recovery from mpox, due to the natural immunity conferred by infection.

PEP vaccination strategies have demonstrated limited effectiveness with an estimated VE of 20% (95%CI −20-65%), after adjusting for immortal time bias, according to a meta-analysis by Pischel et al. This modest effectiveness may be partially explained by delays in vaccine administration, with a median interval of seven days between exposure and vaccination in real-world settings [171, 182].

### Isolation and hygiene

Along with vaccination, infection control strategies are critical to preventing the spread of the disease. These strategies rely on the prompt identification and appropriate isolation of patients, the use of adequate PPE by healthcare workers, and close monitoring of contacts during the incubation period. In 2024, the CDC updated its guidelines on mpox prevention and control in both community and healthcare settings [183, 184]. Patients with suspected or confirmed mpox should be placed in a single-occupancy room with a dedicated bathroom. Movement outside the room should be restricted to medically essential purposes, and visitation limited to individuals essential to patient care. Although airborne transmission of mpox remains under debate, the CDC recommends that healthcare professionals entering the room wear a NIOSH-approved N95 filters respirator, but no special air handling in the room. Recommended PPE also includes gloves, gowns, and eye protection. Poxviruses are known for their prolonged environmental persistence due to resistance to desiccation and high tolerance to temperature and pH, and MPXV transmission via contaminated fomites and lesion material is well documented. Therefore, cleaning of hospital rooms, homes and soiled laundry must be conducted in such a way as to limit the dispersion or aerosolization of infectious materials, using wet cleaning methods, including disinfection with a 0.1% sodium hypochlorite solution, avoiding activities such as dry dusting or vacuuming, and washing linens at a minimum of 60 °C. Patients isolated at home should avoid close contact with others and pets, use a separate bathroom if feasible, minimize sharing of potentially contaminated items (e.g., bed linens or towels) and refrain from sexual activities involving direct physical contact.

Isolation measures must be maintained until all lesions have crusted, those crusts have separated, and a fresh layer of healthy skin has formed underneath, whether in healthcare facilities or at home.

Contact cases (including healthcare professionals) should monitor for signs and symptoms for 21 days following the last known exposure, including a thorough skin exam. They may continue daily activities without specific protective measures except limiting biological donations and engagement in sexual activities.

### Behavioral changes

Data collected from 824 US cisgender MSM in August 2022 revealed that nearly half of the interviewees reported some behavioral changes in response to the 2022 mpox outbreak. These included actively reducing their number of sexual partners, limiting one-time sexual encounters, and reducing sexual activity with partners met via dating apps [185]. Similar findings were reported by Hubach et al. among MSM and transgender individuals in the US in 2022 [186]. Subsequent modeling analyses suggested that these behavioral changes may have played a substantial role in reducing MPXV transmission among MSM, especially through the reduction of one-time partnerships [187].

## Conclusion

Mpox has transitioned from a neglected zoonosis confined to Central and West Africa into a global health threat, with recent outbreaks reshaping our understanding of its transmission dynamics, clinical features, severity, and at-risk populations. This shift has been associated with the emergence of distinct MPXV clades – including clade IIb responsible for the 2022 global outbreak, and clade Ib which emerged in the DRC in 2023 – both characterized with sustained human-to-human transmission and primarily driven by sexual contact, a feature never observed with historical clades. They also differ from previous outbreaks in their clinical presentation and severity, with clade IIb associated with milder disease, and clade Ib appearing to cause less severe disease than the endemic Central African clade Ia. These variations likely reflect a combination of viral genetic factors, host-related characteristics (e.g., demographic and immunological profile of affected populations), and differences in transmission routes.

Although most infections are self-limiting, severe disease can occur, particularly children, pregnant women, and in immunocompromised individuals, such as those with advanced HIV infection. Treatment continues to rely primarily on supportive care. Tecovirimat remains the first-line therapy for severe mpox, although recent clinical trials have failed to demonstrate its efficacy, underscoring the urgent need for continued research into effective antiviral agents. Preventive strategies have benefited from the MVA-BN vaccine, which has demonstrated a favorable safety and efficacy profile, including in immunocompromised individuals, although real-world data on its effectiveness remains limited. However, its impact remains undermined by significant inequities in vaccine access in endemic regions – particularly Western and Central African countries, where the burden of mpox is highest – due to logistical barriers, structural inequalities in global health governance, vaccine supply shortages, and funding constraints.

Looking forward, a coordinated, inclusive, and effective global response will be essential to containing future mpox outbreaks. In this effort, addressing the stigma associated with mpox must be a central priority. Beyond its impact on individuals, such stigma impedes timely diagnosis, discourages care-seeking, and undermines contact tracing, complicating epidemiological studies and outbreak control. It also reinforces the long-standing invisibility of endemic countries in global health agendas, limiting their access to research funding, treatment, and vaccines. Finally, this coordinated global effort must also involve enhancing diagnostic and therapeutic capacities, tackling the social determinants that increase vulnerability to mpox, and ensuring equitable access to preventive tools for those most at risk.

## Data Availability

No datasets were generated or analysed during the current study.
